# Immunogenicity of *Leishmania*-derived hepatitis B small surface antigen particles exposing highly conserved E2 epitope of hepatitis C virus

**DOI:** 10.1186/s12934-016-0460-4

**Published:** 2016-04-13

**Authors:** Anna Czarnota, Jolanta Tyborowska, Grażyna Peszyńska-Sularz, Beata Gromadzka, Krystyna Bieńkowska-Szewczyk, Katarzyna Grzyb

**Affiliations:** Laboratory of Virus Molecular Biology, Intercollegiate Faculty of Biotechnology UG-MUG, University of Gdańsk, A. Abrahama 58, 80-307 Gdańsk, Poland; Laboratory of Recombinant Vaccines, Intercollegiate Faculty of Biotechnology UG-MUG, University of Gdańsk, A. Abrahama 58, Gdańsk, 80-307 Poland; Tri-City Academic Laboratory Animal Centre, Medical University of Gdańsk, Dębinki 1, Gdańsk, 80-211 Poland

**Keywords:** Hepatitis C virus (HCV), HBV small surface antigen (sHBsAg), VLP, *Leishmania tarentolae*, Vaccine

## Abstract

**Background:**

Hepatitis C virus (HCV) infection is a major health problem worldwide, affecting an estimated 2–3 % of human population. An HCV vaccine, however, remains unavailable. High viral diversity poses a challenge in developing a vaccine capable of eliciting a broad neutralizing antibody response against all HCV genotypes. The small surface antigen (sHBsAg) of hepatitis B virus (HBV) has the ability to form highly immunogenic subviral particles which are currently used as an efficient anti-HBV vaccine. It also represents an attractive antigen carrier for the delivery of foreign sequences. In the present study, we propose a bivalent vaccine candidate based on novel chimeric particles in which highly conserved epitope of HCV E2 glycoprotein (residues 412–425) was inserted into the hydrophilic loop of sHBsAg.

**Results:**

The expression of chimeric protein was performed in an unconventional, *Leishmania tarentolae* expression system resulting in an assembly of particles which retained immunogenicity of both HCV epitope and sHBsAg protein. Direct transmission electron microscopy observation and immunogold staining confirmed the formation of spherical particles approximately 22 nm in diameter, and proper foreign epitope exposition. Furthermore, the sera of mice immunized with chimeric particles proved reactive not only to purified yeast-derived sHBsAg proteins but also HCV E2 412–425 synthetic peptide. Most importantly, they were also able to cross-react with E1E2 complexes from different HCV genotypes.

**Conclusions:**

For the first time, we confirmed successful assembly of chimeric sHBsAg virus-like particles (VLPs) in the *L. tarentolae* expression system which has the potential to produce high-yields of properly N-glycosylated mammalian proteins. We also proved that chimeric *Leishmania*-derived VLPs are highly immunogenic and able to elicit cross-reactive antibody response against HCV. This approach may prove useful in the development of a bivalent prophylactic vaccine against HBV and HCV and opens up a new and low-cost opportunity for the production of chimeric sHBsAg VLPs requiring N-glycosylation process for their proper functionality and immunogenicity.

## Background

Although recent advances in hepatitis C treatment give reasons for optimism, a prophylactic vaccine for hepatitis C virus (HCV) remains an elusive goal. The recently developed direct-acting antiviral drugs (DAAs) have improved the sustained viral response (SVR) rates up to even 99 % for Sofosbuvir/Ledipasvir therapy [1]. Unfortunately, the cost of the DAA therapy is still extremely high [2]. Considering the above, the development of an effective prophylactic vaccine against HCV should be a medical priority. HCV infection affects an estimated 2–3 % of human population and the number is even higher in undeveloped countries, including Egypt with the HCV prevalence of 20 % [3]. Approximately 70–80 % of the infected individuals develop chronic liver disease, which can lead to cirrhosis and liver carcinoma.

HCV is a member of the *Flaviviridae* family. Its single stranded positive-sense RNA genome codes for both structural and non-structural viral proteins. The HCV nucleocapsid is surrounded by E1E2 envelope glycoproteins embedded in a lipid envelope. E1 and E2 glycoproteins constitute a potential target for the development of a prophylactic HCV vaccine, as they are involved in virus–host interaction, and the antibodies directed against these proteins seem to neutralize HCV [4]. Due to the fact that resolution of HCV infection is mediated not only by a broad and potent T cell response [5], but also by the neutralizing antibodies (nAbs) raised mainly against HCV E1E2 heterodimer [6, 7], a prophylactic vaccine consisting of adjuvanted recombinant E1E2 heterodimer was proposed. The most advanced approach included immunization with E1E2 complex expressed in Chinese hamster ovary (CHO) cell line. Phase I clinical trials indicated that immunization with glycosylated envelope proteins resulted in potent nAbs and CD 4^+^ T-cell responses [8, 9].

The major obstacle in the development of a protective immunity against HCV is its high genetic diversity and variability. In recent studies, HBV capsid-like particles (CLPs) were used to present variants of the HCV E2 glycoprotein hyper-variable region 1 (HVR1). HVR1 is one of the most immunogenic regions of glycoprotein E2, but its constant evolution and diversity along HCV genotypes leads to limited cross-reactivity of the elicited antibodies [10]. The region located downstream of HVR1 contains a potent and highly conserved epitope first identified by the mouse monoclonal antibody AP33. The region, spanning residues 412–423 of glycoprotein E2, can elicit broadly nAbs capable of inhibiting HCV, both in vitro and in vivo [11, 12]. Epitope AP33 is highly conserved among over 5500 E2 sequences in the GenBank database and mostly regarded as a linear epitope [11]. These features make 412–423 residues a perfect peptide antigen expressed on various antigen carriers. Moreover, the region is only 13 amino acids long and does not include any additional cysteine residue that could result in formation of non-authentic disulfide bonds and disrupt the carrier structure [13]. In general, peptide vaccines used in isolation are weakly immunogenic and require some carries for delivery [14]. This finds support in a recently published report which shows that monoclonal antibodies (mAbs) generated against a cyclic variant of the AP33 epitope bind poorly to E2 and do not neutralize the virus [15].

Yeast-derived HBV small surface antigen (sHBsAg) forms particles 22 nm in diameter currently used worldwide as the commercial recombinant hepatitis B vaccine. sHBsAg tertiary structure forms a highly conserved, hydrophilic loop containing the major B-cell epitopes also known as the “a”-determinant [16, 17]. Because of its immunogenic potential, sHBsAg was also applied as an antigen carrier to deliver foreign sequences and induce anti-foreign humoral and cellular responses [13, 18–25].

The present study focused on construction, characterization and immunological studies of novel sHBsAg chimeric particles produced in the *L. tarentolae* expression system. The system enables production of recombinant proteins with their mammalian-like N-glycosylation pattern. Moreover, *L. tarentolae* can grow in biofermenters to a high cell density and the recombinant protein production yield can reach several milligrams per liter of culture [26, 27]. Here, we propose a new vaccine candidate based on chimeric particles in which the HCV E2 glycoprotein region (aa 412–425) is inserted within the “a”-determinant loop of sHBsAg. We show that *Leishmania*-derived HBV/HCV chimeric particles elicit promising titers of cross-reactive antibodies against HCV. These particles also induce both humoral and cellular immune response against HBV, opening a new and low-cost approach to bivalent vaccine development.

## Results

### Expression and characterization of sHBsAg and 412–425_sHBsAg particles

**Fig. 1:**
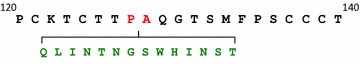
Schematic illustration of the sHBsAg sequence with the foreign HCV E2 412–425 epitope inserted into the “a”-determinant region. The sHBsAg insertion site corresponding to amino acid positions 127 and 128 is marked *red*. The numbering corresponds to the amino acid positions in the sHBsAg protein. The 412–425 epitope of the HCV E2 glycoprotein is shown in *green*

**Fig. 2:**
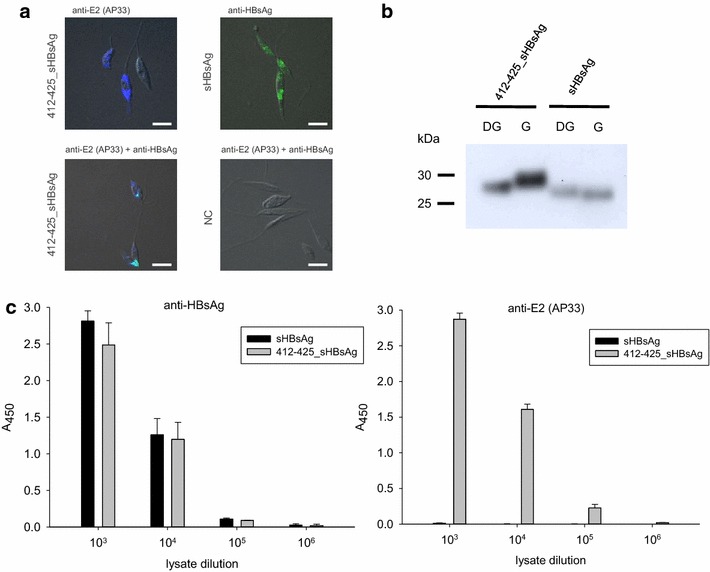
Characterization of the particles expressed in the *L. tarentolae* system. **a** Immunofluorescence of recombinant *L. tarentolae* cells transfected with plasmids expressing 412–425_sHBsAg and sHBsAg. Cells transfected with empty *pLEXSY_I*-*blecherry3* plasmid were used as negative control (NC). The staining was carried out using AP33 mAbs (*blue*) and anti-HBsAg Abs (*green*), scale bar = 5 µm. **b** Western blot analysis of the *Leishmania*-derived particles in reducing conditions. sHBsAg and 412–425_sHBsAg were treated with PNGase F and detected using the anti-HBsAg specific antibody. G represents the glycosylated and DG deglycosylated form of protein. **c** Recognition of particles with anti-HBsAg and AP33 Abs in the ELISA tests. ELISA plates were coated with serial dilutions of recombinant *L. tarentolae* cell lysates containing sHBsAg and 412–425_sHBsAg particles. The dilution factor is depicted on *x*-axis. For each ELISA assay, the mean from three independent experiments performed in duplicate is shown. The mean A_450_ values and standard deviations are shown on the *y*-axis. The background from the *L. tarentolae* wild-type cell lysate in each dilution was subtracted from the obtained results

The HCV E2 glycoprotein region spanning 412–425 residues was chosen as the epitope of broadly neutralizing properties [28] and inserted into the major antigenic loop of HBV sHBsAg protein (Fig. 1). The expression of the recombinant proteins was carried out in high-density cell cultures of recombinant parasites, using tetracycline-inducible *L. tarentolae* expression system. The expression of proteins was confirmed by immunofluorescence (Fig. 2a), western blot (Fig. 2b) and ELISA (Fig. 2c) with protein-specific antibodies: anti-HBsAg and anti-E2 (AP33). The confocal studies indicate that both proteins are predominantly located in the cytosol of *L. tarentolae* cells, probably in endoplasmic reticulum (ER). No particle secretion into the culture medium was observed. The western blot analysis of cell lysates showed that in reducing conditions, the molecular masses of monomers of sHBsAg and 412–425_sHBsAg were approximately 27 and 30 kDa, respectively (Fig. 2b). The difference in the molecular masses of the monomers is probably associated not only with the insertion of the 14 aa long fragment but also with the molecular mass of the N-glycans. The chimeric protein 412–425_sHBsAg contains two additional N-glycosylation sites at positions 417 and 423 versus the wild-type sHBsAg. N-glycosylation of 412–425_sHBsAg was confirmed by reaction with PNGase F, where a decrease in the protein molecular weight after endoglycosidase digestion was observed (Fig. 2b). In ELISA test, 412–425_sHBsAg chimeric particles were specifically and efficiently recognized by neutralizing AP33 mAb with no cross-reactivity with sHBsAg particles. 412–425_sHBsAg particles were also recognized by polyclonal anti-HBsAg antibodies at the efficacy similar to that of sHBsAg (Fig. 2c).

**Fig. 3:**
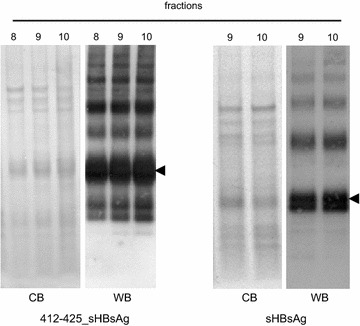
OptiPrep density gradient centrifugation of recombinant particles. Seventeen fractions of 0.5 mL each were harvested from the top of the OptiPrep gradient after centrifugation. The aliquots of each fraction were analyzed by SDS-PAGE Coomassie blue staining (CB) and western-blot (WB) with anti-HBsAg Abs. The figure represents the results of fractions 8–10 where the highest amount of particles was detected

**Fig. 4:**
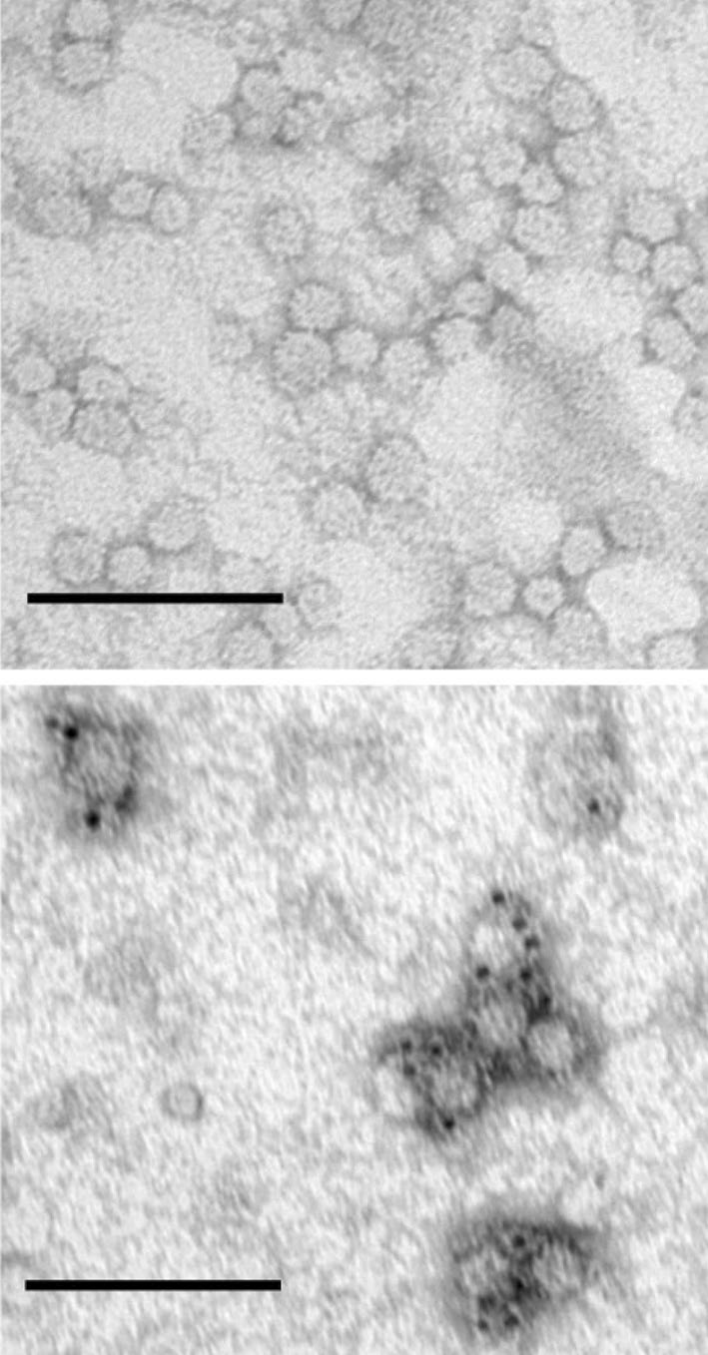
Electron micrograph of chimeric 412–425_sHBsAg particles. After centrifugation on OptiPrep gradient, the particles were negatively stained with uranyl acetate and analyzed by electron microscopy (*top*). Numerous particles approximately 22 nm in diameter were observed. The exposition of the 412–425 epitope was studied by immunogold labeling using AP33 mAbs and secondary goat anti-mouse Abs conjugated with 6 nm gold particles (*bottom*). *Scale bar* 100 nm

The sHBsAg and 412–425_sHBsAg particles were partially purified from cell lysate by ultracentrifugation on OptiPrep gradient, and 0.5 mL fractions were harvested and analyzed by western blot using anti-HBsAg antibodies. The purity of the fractions was estimated with Coomassie stained SDS-PAGE gel (Fig. 3). Multimers of higher molecular mass were also detected for both proteins. Fractions with the highest concentration of particles were pooled, and protein concentration measured by Bradford assay reached 15–20 mg/L of the induced cell culture. Interestingly, one-step purification process of the particles on OptiPrep gradient proved effective. Finally, electron microscopy analysis of the 412–425_sHBsAg fraction showed spherical-particles approximately 22 nm in diameter, with morphology resembling the icosahedral structures of *P. pastoris*-derived particles [29]. Additionally, immunogold labeling of 412–425_sHBsAg particles with AP33 antibodies specific to the 412–425 epitope revealed selective labeling of 22 nm particles, which suggests strongly that the 412–425 epitope is properly exposed on the surface of sHBsAg particles (Fig. 4). In summary, these results confirm an efficient expression of both sHBsAg and 412–425_sHBsAg proteins as well as a proper exposition of the 412–425 epitope on sHBsAg carrier.

### Immunogenicity of sHBsAg and 412–425_sHBsAg particles

**Fig. 5:**
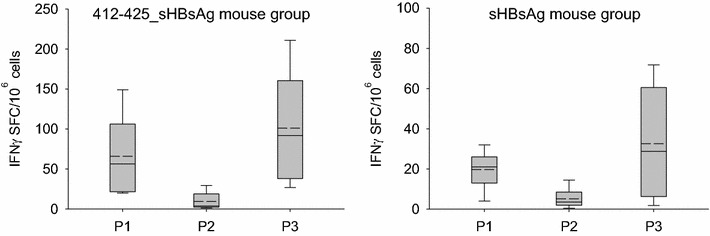
Analysis of cellular response in BALB/c mice immunized with *Leishmania*-derived particles. *Box plot* of antigen-specific IFN-γ ELISPOT responses in sHBsAg (*right*) and 412–425_sHBsAg (*left*) groups of immunized mice. Splenocytes from six immunized BALB/c mice per group were stimulated with 20 µg/well of 43 different 15-mer overlapping peptides spanning the sHBsAg protein sequence pooled into three groups (P1, P2, P3). The splenocytes were collected 2 weeks after the last immunization. The *gray region* is the 25–75th percentile; the *horizontal lines* indicate the mean (*dashed*) and median (*solid*) values

To demonstrate immunogenicity of sHBsAg and chimeric 412–425_sHBsAg particles, two groups of BALB/c mice were immunized subcutaneously on days: 0, 14, and 28 with fractions containing sHBsAg or 412–425_sHBsAg particles. All mice were immunized in presence of a squalene-based oil-in-water nanoemulsion adjuvant. Two weeks after the last vaccination, splenocytes were isolated and T cell response was analyzed by gamma interferon (IFN-γ) enzyme-linked immune spot (ELISPOT) assay. For splenocytes stimulation we designed peptides covering the entire sHBsAg protein sequence, 15 amino acids in length with an overlap of 10. The peptides were divided into three pools corresponding to amino acid positions: 1–99 (P1 n = 19), 100–188 (P2 n = 16) and 189–240 (P3 n = 9). IFN-γ ELISPOT data were found comparable for both antigens, however a more potent cellular response was noted for 412–425_sHBsAg. The strongest T-cell response was observed for pools 1 and 3 (Fig. 5).

**Fig. 6:**
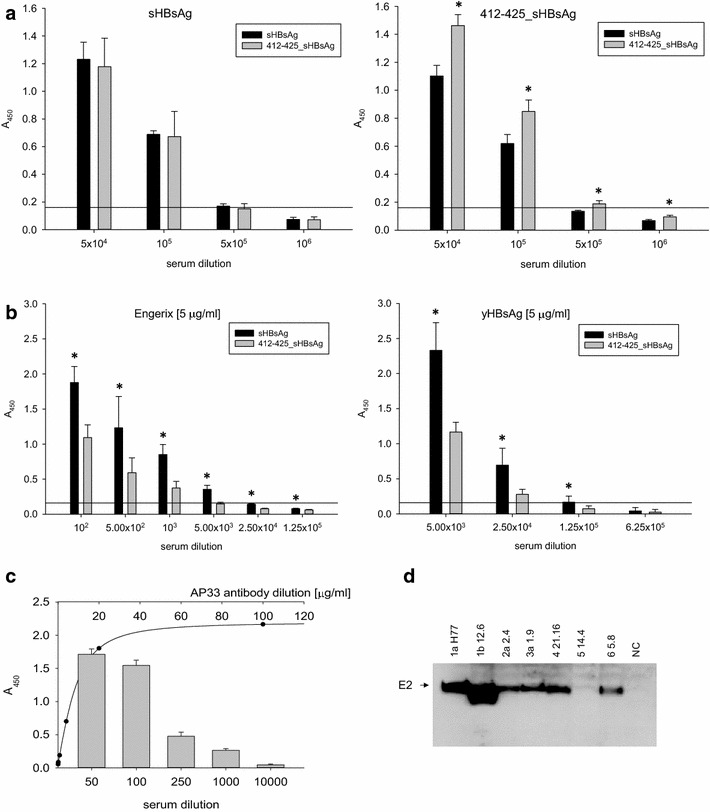
Analysis of the humoral response induced by *Leishmania*-derived particles in BALB/c mice. **a** Analysis of the antibody endpoint titers of the pooled mouse antisera specific to the recombinant particles. ELISA plates were coated with 412–425_sHBsAg (*right*) or sHBsAg (*left*) particles. **b** Analysis of the interaction of immune sera with yeast-derived HBsAg proteins. ELISA plates were coated with 5 µg/mL of purified HBsAg protein from *P. pastoris* (yHBsAg) (*right*) or commercially available vaccine against HBV (Engerix-B) (*left*). **c** Analysis of the antibody response to the 412–425 synthetic peptide. ELISA plates were coated with 20 µg of 412–425 peptide. AP33 mAb was used to estimate concentration of Abs specific to the 412–425 region in serum. Dilution factor of the pooled 412–425_sHBsAg sera and concentration of the AP33 antibody are shown on *x*-axis (*bottom* and *top*, respectively). The 412–425_sHBsAg sera values are represented with *bars*, the AP33 values are marked with *solid line*. Mean A_450_ values and standard deviations are shown on the *y*-axis. The background from the negative control serum in each dilution was subtracted from the obtained results (**a**, **b**, **c**). For each ELISA assay, the mean from three independent experiments performed in duplicate is shown. *Asterisks* indicate statistical significance (*P < 0.05, paired two-tailed *t*-test) (**a**, **b**). The solid horizontal line (**a**, **b**) indicates the cutoff value (three times the mean background value). **d** Analysis of cross-reactivity of the 412–425_sHBsAg sera to the E1E2 complex from different HCV genotypes. The figure represents western blotting in reducing conditions with 412–425_sHBsAg sera diluted 1:500. As an antigen, extracts of HEK293 cells transfected with plasmids expressing E1E2 glycoproteins from different HCV genotypes were used. Non-transfected HEK293 cell lysate was used as negative control (NC)

Two weeks after the last immunization blood samples were collected and the antibody titers determined. Pooled sera reacted strongly to the antigens used for immunization, with the endpoint titer reaching as high as 5 × 10^5^ (Fig. 6a). Weaker antibody response was observed for 412–425_sHBsAg particles when detected with sera from sHBsAg immunized mice (Fig. 6a right). Detailed serum characterization showed cross-reactivity with both purified yeast-derived HBsAg protein (yHBsAg) and a commercially available HBV vaccine (Engerix-B) (Fig. 6b). The results proved that sera from both groups of mice contained high titers of anti-sHBsAg antibodies, and this suggests that 14 aa foreign epitope insertion within sHBsAg particle does not interfere dramatically with the humoral response against sHBsAg protein itself. Similar induction of anti-HBsAg antibodies was observed in mice immunized with chimeric HBsAg particles which delivered HCV-specific cysteine-containing sequence [13]. However, the immune response against sHBsAg protein was lower in the 412–425_sHBsAg group of mice compared to the sHBsAg wild-type group. Of note, the absorbance values obtained for the purified yHBsAg protein were higher than those obtained from the vaccine (Fig. 6b). This can be caused by the fact that sHBsAg particles in vaccines are associated to aluminium hydroxide which can lower test sensitivity due to epitope coverage [30].

Next, we studied the antibody response of 412–425_sHBsAg particles to 412–425 epitope. Interestingly, sera from the 412–425_sHBsAg group were able to recognize not only the sHBsAg protein (as mentioned above), but also synthetic peptide corresponding to the 412–425 region of HCV E2 glycoprotein. The endpoint titer in peptide ELISA test reached 10^3^. The AP33 standard curve showed that the amount of anti-412–425 epitope antibody in serum corresponded to AP33 antibody concentration of approximately 0.9 mg/mL (Fig. 6c). Furthermore, we decided to test cross-reactivity of the sera from 412–425_sHBsAg immunized mice against E1E2 complex from different HCV genotypes. As expected, the antisera recognized E2 glycoproteins from HCV genotypes 1a, 1b, 2b, 3a, 4, and 6, but failed to recognize E2 derived from genotype five (Fig. 6d). That was due to the fact that genotype five isolate had 4 amino acid changes in region 412–423 in comparison with the sequence used to create 412–425_sHBsAg protein. Such a modification may influence epitope recognition as well as neutralization of the HCV pseudotype particles (HCVpp) [11]. All facts considered, the 412–425_sHBsAg sera were found broadly cross-reactive across the HCV genotypes.

To recapitulate, the immunization studies confirmed that both *Leishmania*-derived particles are highly immunogenic in mice. Moreover, the 412–425_sHBsAg sera recognized efficiently both the 412–425 peptide and mammalian cell culture-derived E2 protein from different HCV genotypes, as well as yeast-derived sHBsAg proteins.

## Discussion

Despite the discovery of highly effective antiviral agents, 3–4 million new HCV infections every year still represent a major health problem. Therefore, the development of an effective and a low-cost prophylactic vaccine is needed to control global HCV infection. For years, humoral response and nAbs were considered to play a side role in HCV infection process. However, recent studies suggest that nAbs play an active role in HCV spontaneous resolution [6, 7, 31]. During the infection, all known nAbs target epitopes within the HCV envelope glycoproteins E1 and E2, prevailingly the E2 epitopes. The ectodomain of the E2 glycoprotein contains three regions of variability that are targeted by nAbs—HVR1, HVR2, plus the intergenotypic variable region. HVR1 is a 26–28 aa long fragment responsible for interactions with cellular SR-BI receptor [32]. Because of its high immunogenicity, HVR1 was often considered a potential vaccine epitope. However, a recent approach where HVR1 is used as an antigen carried on HBV capsid-like particles showed deficiency in the HCVpp and cell culture HCV (HCVcc) cross-neutralization [10]. Unfortunately, HVR1 is the most variable region of HCV and the high rate of mutation within this region results in poor cross-neutralization across different HCV isolates. It has been reported that HVR1 plays the role of a major epitope decoy that elicits isolate-specific nAbs from which the HCV can easily escape [33].

It has previously been demonstrated that CD81-binding regions of HCV E2 glycoprotein are highly conserved and represent the main target for the broad range of nAbs [11, 31, 34, 35]. Thus, the intrinsically conserved epitopes able to induce cross-neutralizing antibodies seem to be essential in vaccine development. The recent data suggest that recombinant E1E2 complex is capable of eliciting antibodies that bind the conserved residues overlapping epitopes of many broadly cross-neutralizing antibodies targeting E2-CD81 interaction [36].

In this study, we decided to design particles carrying broadly-neutralizing 412–425 epitope and insert it into the major antigenic loop of sHBsAg protein. Both sHBsAg (wild-type) and 412–425_sHBsAg (chimeric) proteins were expressed in *L. tarentolae* cells at similar efficiencies. The fact indicates that epitope insertion did not impair the protein folding. This is consistent with observation from previous study which showed that “a”-determinant of sHBsAg can support 36 amino acids insertions without an impact on the particle expression efficiency [23]. The insertion of 412–425 epitope in “a”-determinant was confirmed by cell lysate ELISA and immunogold labeling of particles with AP33 antibodies. The EM analysis revealed that 412–425_sHBsAg particles were present in the cell lysate and their shape resembled the correctly assembled sHBsAg particles expressed in the mammalian or yeast expression systems [29, 37].

Over the last few years, new vaccination approaches involving VLP-based vaccines have been developed. VLP are effective immunogens and due to their highly organized structure they are able to elicit both the nAbs and T-cell responses [38]. sHBsAg has been shown to self-assemble into highly immunogenic, non-infectious subviral particles, and is used worldwide as a safe, commercial hepatitis B vaccine. The sHBsAg protein was previously expressed in mammalian [37], baculovirus [39], *E.coli* [40], and yeast systems utilizing *P. pastoris* [29, 41]*, S. cerevisiae* [42]*, H. polymorpha* [37]*, and Y. lipolytica* [43]. The choice of *S*. *cerevisiae* yeast for commercial production of sHBsAg was dictated by low cultivation costs and high protein yield. However, unlike in the mammalian expression system, the yeast-derived sHBsAg is not glycosylated, which is a major drawback of the yeast-based systems. Noteworthy, sHBsAg expression in mammalian cells leads to particle secretion [18, 23, 44]. The process significantly simplifies purification of particles from the culture medium. On the other hand, particle secretion was never observed in yeast systems where protein was retained within ER partially assembling into multi-layer lamellar structures. There is no evidence for the presence of the particles within the yeast cells [29]. Importantly, the assembly of *L. tarentolae* expressed particles was confirmed by electron microscopy, though we never observed any particle secretion into the culture medium. They are possibly retained in ER, just as the yeast-derived virus-like particles (VLPs), and the assembly of the particles probably occurs after the cell lysis in the subsequent down-stream processing.

This report is the first to describe efficient expression of sHBsAg in a eukaryotic protozoan *L. tarentolae*-based expression system. The *L. tarentolae* system has recently been used to express immunogenic human papillomavirus (HPV) L1 VLPs [45] and to produce recombinant proteins with the N-glycosylation pattern similar to the mammalian one, as demonstrated for erythropoietin [26] and soluble amyloid precursor protein alpha (sAPPalpha) [46]. Region 412–425 of the HCV E2 glycoprotein includes two highly conserved glycosylation sites which have recently been shown to play an important role in epitope recognition by the broadly neutralizing monoclonal antibodies AP33, HCV1 [47], and HC33.11 [48]. Moreover, it has previously been shown that the structure of oligosaccharides attached to hemagglutinin can play an important role in its immunogenicity [49]. The N-glycosylation pattern of *L. tarentolae*-derived particles should be further analyzed, although higher mass of the 412–425_sHBsAg protein and the results of deglycosylation with PNGase F suggest the presence of additional N-glycans.

Recently, various strategies of using sHBsAg as the prophylactic HCV vaccine candidates have been investigated. It has been shown that pre-existing immunity to sHBsAg does not impede immune response against foreign epitopes or proteins carried on an sHBsAg particle. This suggests that chimeric HBsAg-based particles can be used in the booster dose strategy in individuals previously vaccinated against HBV [50, 51]. The use of sHBsAg particles as carriers of small HCV epitopes inserted into the antigenic external hydrophilic loop was reported in several studies [23, 24, 52]. Another approach involved the design of chimeric HBV-HCV particles in which the N-terminal transmembrane domain (TMD) of the sHBsAg protein was replaced with the TMD of HCV full-length E1 or E2 proteins [19]. Those chimeric particles expressed in CHO cells elicited strong antibody response against both HBsAg and HCV envelope proteins in immunized rabbits [53]. Moreover, the sera displayed cross-neutralizing activity against HCVpp and HCVcc, although the T-cell response of chimeric particles was not analyzed. Here, we tested the ability of *Leishmania*-derived particles to elicit a cellular response using an in vitro T cell activation assay. The strongest response occurred against sHBsAg peptide pools one and three. These results are in agreement with other reports suggesting that most T-cell immunodominant epitopes are located outside the “a”-determinant region [54, 55]. It also proves that the location of a foreign epitope does not interfere with cellular response against chimeric VLPs. Not surprisingly, no cellular response was detected when splenocytes were stimulated with 412–425 synthetic peptide (the results not shown). The outcome supports the previous findings, namely that almost no cellular epitopes were present on HCV envelope glycoproteins versus the core or NS proteins [5, 56]. Additionally, we observed a higher cellular response in mice immunized with chimeric 412–425_sHBsAg particles than in those immunized with wild-type particles. An N-glycan structural analysis confirmed that *L.tarentolae* is able to produce mammalian-like, as well as mannose-terminated N-glycans [26]. Several reports show that mannosylation of particles enhances antigen uptake and processing by the antigen presenting cells [57–59]. Possibly, additional N-glycans present in 412–425_sHBsAg particles are mannose-terminated and may promote a stronger cellular response. Nevertheless, it should be emphasized that no significant differences in the humoral responses were observed.

The antibody response to mammalian and yeast-derived sHBsAg is well documented [37, 60]. In this study, we proved strong immunogenicity of *L. tarentolae*-derived particles, since the antibody titers in the sera of both analyzed mouse groups reached 5 × 10^5^ (Fig. 6a). The 412–425_sHBsAg sera were able to detect both synthetic peptide (Fig. 6c) and yeast-derived sHBsAg proteins (Fig. 6b). However, immune response against sHBsAg protein was weaker in the 412–425_sHBsAg group of mice than in the sHBsAg wild-type group. This is consistent with the previous findings which described lessening of the anti-sHBsAg immune response in animals immunized with chimeric particles versus those immunized with wild-type particles [24]. It has also been demonstrated that changes within external hydrophilic loop are strongly associated with the sHBsAg protein antigenicity [61]. Furthermore, our findings proved that 412–425_sHBsAg sera were able to recognize not only the synthetic peptide, but also HCV E2 glycoprotein of most virus genotypes, which indicated presence of broadly cross-reactive antibodies (Fig. 6d).

There is abundant evidence clearly demonstrating the importance of the cross-neutralizing antibodies in spontaneous clearance of HCV infection. Promising results have been obtained for adenovirus-based vaccine proved to induce a broad, strong and long-lasting T-cell response in healthy humans [62]. Thus, the development of a prophylactic vaccine can be achieved by combination of antigens which are able to elicit simultaneous T and B cell responses, both crucial for the successful protection against HCV [10, 36, 63].

## Conclusions

Since HCV displays high genetic diversity, the ideal prophylactic vaccine should elicit antibodies against the highly conserved viral regions. Our results indicate that chimeric sHBsAg VLPs carrying a highly conserved HCV epitope may elicit cross-reactive antibodies against HCV while preserving anti-sHBsAg “a”-determinant immunogenicity. This approach, combined with a vaccine inducing a strong T-cell response against HCV, may be useful in developing a cost-effective bivalent prophylactic vaccine against the two main hepatotropic human pathogens. Moreover, chimeric sHBsAg particles were successfully expressed in *L.tarentolae* system, which can be considered an alternative to mammalian cells for large-scale production of N-glycosylated VLPs for pharmaceutical purposes.

## Methods

### Plasmids

Figure 1 summarizes the construction of the 412–425_sHBsAg chimeric gene used in this work. The region of HCV E2 glycoprotein, spanning the 412–425 residues (isolate H77c, GenBank accession no. AF011751) was inserted within HBV subtype adw2 sHBsAg amino acid positions 127P and 128A (GenBank accession no. AF397207.1). The construct was obtained by gene synthesis using *L. tarentolae*-adapted codon (GeneArt—Thermo Fisher Scientific). The gene coding for sHBsAg was synthetized as mentioned above. Synthetized genes were ligated into BglII—NotI restriction sites in the *pLEXSY_I*-*blecherry3* vector (Jena Bioscience). For HCV E2 glycoprotein expression in human epithelial kidney (HEK) 293 cells, we used plasmids coding for full-length E1E2 derived from HCV genotypes: 1a (isolate H77.20); 1b (UKN1B.12.6); 2a (UKN2A.2.4); 3a (UKN3.1.9); 4 (UKN4.21.16); 5 (UKN5.14.4); and 6 (UKN6.5.8) – kindly provided by J. Ball.

### *Leishmania tarentolae* cultivation and protein expression

HBV small surface antigen and 412–425_sHBsAg proteins were expressed using the inducible LEXSY expression system according to the manufacturer’s instructions (Jena Bioscience). Briefly, the plasmids were transfected into *L. tarentolae* cells by electroporation. Transfected cells were selected with bleomycin (100 µg/mL) in suspension culture. Subsequently, recombinant cell lines were cultivated in 25 cm^2^ tissue culture flasks filled with 10 mL of selective medium supplemented with hemin at 26 °C and kept away from light. The T7 promoter driven transcription was induced by adding tetracycline to the final concentration of 15 µg/mL. Cells were grown in 500 mL shake flasks for 72 h, at 26 °C, in agitated culture to the final optical density of 4–5 at 600 nm (OD_600_).

### Indirect immunofluorescence

For immunofluorescence labeling, tetracycline-induced *L. tarentolae* cells were washed with PBS and fixed in 4 % paraformaldehyde for 30 min at RT. Lysine-coated glass coverslips were covered with fixed cell suspension and left to dry at RT. Next, the cells were permeabilized with 0.2 % Triton X-100 in PBS for 10 min at RT. Subsequently, the coverslips were incubated with primary rabbit anti-HBsAg (Abcam) and mouse anti-E2 AP33 (kindly provided by A. Patel) antibodies diluted to 1:1000 in PBS-BSA buffer [0.5 % (w/v) BSA] for 1 h at RT. The coverslips were then washed with PBS and incubated with Alexa Fluor 633-labeled goat anti-mouse and Alexa Fluor 488-labeled goat anti-rabbit secondary antibodies (1:1000 in PBS-BSA) for 1 h at RT. After washing, the coverslips were mounted onto microscope slides with ProLong Gold antifade reagent.

### SDS-PAGE and western blot

Analysis of the particle expression was carried out by SDS-PAGE using 4–12 % gradient Bis–Tris gels in MES SDS running buffer. After electrophoresis, proteins were transferred onto PVDF membrane using electroblotting, and subsequently the membranes were blocked overnight at 4 °C with 3 % nonfat milk in TBST [TBS buffer, 0.1 % (v/v) Tween-20]. Following blocking, membranes were incubated for 1 h at RT with primary anti-HBsAg antibodies diluted in TBST, washed with TBST, and then incubated with goat anti-rabbit secondary AP—conjugated antibodies (Santa Cruz Biotechnology). The results were developed using the BCIP/NBT substrate.

For testing serum cross-reactivity, HEK 293 cells were transfected with plasmids expressing glycoproteins E1E2 derived from different HCV genotypes. 72 h after transfection, cells were washed with PBS buffer and lysed in lysis buffer (PBS buffer, 1 % Triton X-100). The clarified cell lysates were separated using SDS-PAGE and transferred onto PVDF membrane as described above. Following the blocking, the membranes were incubated for 1 h at RT with a mixture of pooled 412–425_sHBsAg sera diluted 1:500, washed with TBST and then incubated with goat anti-mouse secondary HRP—conjugated antibodies (Santa Cruz Biotechnology).

### PNGase F treatment

412–425_sHBsAg and sHBsAg cell lysates were boiled for 10 min in denaturing buffer (0.5 % SDS, 40 mM DTT). The samples were divided into two equal portions and incubated for 16 h at 37 °C, with or without PNGase F, according to the manufacturer’s instructions (New England Biolabs). The results were visualized with western blot using anti-HBsAg antibodies as described above.

### Cell lysis and ultracentrifugation

Hundred milliliter of tetracycline-induced cell culture was centrifuged at 4 °C, 8000 rpm, for 15 min. The cell pellet was either stored at −20 °C until use or immediately resuspended in 10 mL of ice-cold lysis buffer [PBS buffer, 0.6 % (v/v) Tween-20]. Cells were sonicated and the suspension was clarified by centrifugation at 4 °C, 8000 rpm, for 35 min.; the supernatant was left for 16–24 h at RT for particle formation. Subsequently, the lysate was layered on an OptiPrep (Sigma-Aldrich) gradient formed in ultra-clear tubes [2 mL of 30 % (v/v) OptiPrep, 2 mL 24 % (v/v) OptiPrep, 1.5 mL 18 % (v/v) OptiPrep, 1.5 mL 12 % (v/v) OptiPrep, and 1.5 mL 6 % (v/v) OptiPrep in ultra-clear water] and ultracentrifuged at 27,000 rpm for 16 h at 4 °C. Then, 500 µL fractions were harvested and analyzed by western blot using anti-HBsAg rabbit polyclonal antibodies (Abcam). Purity of the fractions was analyzed by SDS-PAGE with Coomassie R-250 staining. sHBsAg positive fractions of the highest purity were pooled, and OptiPrep buffer was replaced with PBS using Amicon Ultra 100 K centrifugal units (Merck Millipore). The thus prepared samples were used for further analysis.

### Cell lysate ELISA

ELISA 96-well plates were left for coating with cell lysates diluted in PBS for 16 h at 4 °C. Each well was then blocked with 3 % (w/v) BSA in PBST [PBS buffer, 0.05 % (v/v) Tween-20] for 2 h at RT. Primary mouse monoclonal AP33 and rabbit polyclonal anti-HBsAg (Acris Antibodies) were diluted in PBST with 0.3 % (w/v) BSA and added to the wells. After the washing, primary antibodies were detected with the goat anti-mouse or goat anti-rabbit secondary HRP-conjugated antibodies (Santa Cruz Biotechnology). The reaction with TMB substrate was stopped with 0.5 M H_2_SO_4_ and signal intensity at 450 nm was measured using plate reader (TECAN).

### Immunization protocol

Groups of 6 BALB/c female mice, 6–8 weeks of age, were immunized subcutaneously with squalene-based oil-in-water nanoemulsion adjuvant (Addavax, InvivoGen). The mice were immunized with 15 µg of protein on day 0, and with 10 µg on days: 14 and 28. The mice used as negative controls were immunized with PBS-adjuvant mixture alone. All experiments on animals were conducted by an accredited company (Tri-City Academic Laboratory Animal Centre, Medical University of Gdańsk), in accordance with the current guidelines for animal experimentation. The protocols were approved by the Committee on the Ethics of Animal Experiments of the Medical University of Gdańsk (Permit Number: 46/2012). All surgery was performed under isoflurane anesthesia, and all efforts were made to minimize suffering.

### Analysis of antibody response

Mouse sera were collected on day 42 and pooled into two groups. Antibody response was measured by direct solid-phase ELISA using immunogens 10 µg/mL, 412–425 synthetic peptide (QLINTNGSWHINST) 20 µg/mL (JPT-Innovative Peptide Solutions), commercial vaccine against HBV 5 µg/mL (Engerix-B, GlaxoSmithKline) or yeast-derived HBsAg protein 5 µg/mL (Acris Antibodies). Plates were blocked for 2 h with 3 % (w/v) BSA in PBST. Following the blocking and washing, serial diluted mouse sera were added to the wells and incubated for 1 h at RT. Diluted primary mouse AP33 mAb were used as reference. Secondary goat anti-mouse or goat anti-rabbit Abs were used for detection.

### Statistical analysis

Statistical analyses were performed using the SigmaPlot 12.0 software (SYSTAT Software).

### ELISPOT assay

T-cell response was analyzed by ELISPOT assay for detection of IFN-γ according to the manufacturer’s instructions (BD Biosciences). Briefly, ELISPOT 96-well plates were coated with purified anti-mouse IFN-γ antibody and blocked using RPMI Medium 1640 (Thermo Fisher Scientific) supplemented with 10 % fetal bovine serum, 100 μg/mL penicillin, and 2 mM l-glutamine. Splenocytes were isolated from the mice 42 days after immunization by passage through 70 μm strainers followed by treatment with ACK lysing buffer (Thermo Fisher Scientific). After washing, splenocytes were seeded at 5 × 10^5^ cells/well and stimulated overnight at 37 °C with overlapping peptides (JPT-Innovative Peptide Solutions) pooled into 3 groups spanning the sHBsAg amino acid sequence at the concentration of 20 μg/mL. Concanavalin A was used as positive control. The mean number of spots from duplicate wells were calculated for each animal and adjusted to represent the mean number of spots per 10^6^ splenocytes.

### Electron microscopy and immunogold labeling

For visualization of the particles, the Optiprep gradient fractions were diluted 1:5 in PBS and deposited on formvar-carbon coated 200 mesh nickel grids. Negative staining was performed using 2 % uranyl acetate. Following the staining, samples were analyzed using transmission electron microscope (University of Gdańsk, Poland). For immunogold labeling, grid-deposited 412–425_sHBsAg particles were fixed using 0.1 % (v/v) glutaraldehyde in 4 % paraformaldehyde. Next, the grids were layered on top of AP33 antibodies diluted 1:20 in incubation buffer [PBS buffer, 0.1 % (v/v) BSA-c], and incubated at 4 °C for 16 h. After incubation with primary antibodies, the grids were washed six times using incubation buffer for 5 min at RT. Labeling was performed with goat anti-mouse IgG conjugated with 6 nm gold particles (Aurion), diluted 1:40 in incubation buffer and left for 1 h at RT. After the washing step, the grids were stained with uranyl acetate, dried and analyzed using transmission electron microscope JEM 1200 EX (JEOL Co., Japan) at the Nencki Institute (Warszawa, Poland).
